# Effects of inoculation with four mycorrhizal species on seed phenolic and fatty acids of sesame plants grown under different irrigation regimes

**DOI:** 10.1038/s41598-023-42375-9

**Published:** 2023-09-30

**Authors:** Masoumeh Ghasemi, Morteza Zahedi, Mahdi Gheysari, Mohammad R. Sabzalian

**Affiliations:** 1 Department of Agronomy and Plant Breeding, College of Agriculture, Isfahan University of Technology, Isfahan, 84156-83111 Iran; 2 Department of Water and Science Engineering, College of Agriculture, Isfahan University of Technology, Isfahan, 84156-83111 Iran

**Keywords:** Microbiology, Plant sciences

## Abstract

This study evaluated the interaction effects of irrigation level (well-watered and water stress conditions) and inoculation by different mycorrhizal species (non-inoculated, *Funneliformis mosseae*, *Rhizophagus irregularis, Claroideoglomus claroideum,* and *Glomus fasciculatum*) on mycorrhizal colonization, antioxidant activity, seed yield and oil quality of two sesame cultivars (Yekta and Naz). Water deficit decreased mycorrhizal colonization, seed yield and oil concentration but increased antioxidant activity and seed total phenol and flavonoid concentrations. However, mycorrhizal inoculation increased antioxidant activity, seed yield, oil concentration and total phenolic and flavonoids. The lowest reduction by water stress and the highest increase by inoculation in seed yield were observed in Naz plants inoculated by *Cl. claroideum*. Principal component analysis showed the highest differentiation effect of water stress compared to mycorrhizal inoculation on both cultivars, indicating the relative sensitivity of the two cultivars to water deficit. However, the application of different species of mycorrhizal fungi versus the non-inoculation conditions was somewhat discriminative. In terms of fatty acids, in most cases, water stress increased oleic, palmitic and stearic acids and decreased linoleic and linolenic acids but inoculation increased oleic and linoleic acids and decreased linolenic, palmitic and stearic acids. Regarding phenolic and flavonoids components, the contents of chlorogenic and caffeic acids were increased by water stress but no consistent trend was noted in response to water stress for the other compounds. Mycorrhizal inoculation generally decreased chlorogenic acid but increased gallic, caffeic, p-coumaric, and ferulic acids. In conclusion, the results of the present study may help to increase the level of valuable compounds in sesame for further pharmaceutical purposes under water stress conditions and mycorrhizal symbiosis.

## Introduction

The usable part of sesame (*Sesamum indicum*) is mainly seeds which contain about 75% fat and protein and are fully used in bread making, cakes and sweets. About 75 to 80% of worldwide produced sesame grain is used for oil extraction^1^. The presence of sesamulin in oil which can be decomposed into an antioxidant substances, called sesamol, increases its quality and stability of sesame oil against heat, a characteristic not seen in other oils^2^. Sesame seeds are also used as a rich source of protein, vitamins and minerals for human and animal nutrition^3^.

Sesame is a relatively drought tolerant plant. However, its seedlings are very sensitive to water deficit and as a result the establishment of sesame is difficult in water shortage areas^4^. This plant is also sensitive to dehydration in early flowering and granulation. If proper cultivars and crop management are used, sesame can be harvested in areas with at least 600 mm of annual rainfall^5^.

Drought stress is an important limiting factor in crop production affecting plant physiological and biochemical responses by changing parameters such as relative water content, proline accumulation, photosynthetic capacity, osmotic regulation, stomatal gas exchange and water use efficiency^1, 6^. During drought stress, stomatal closure for the purpose of moisture retention reduces CO_2_ gas exchange leading to the production of reactive oxygen species (ROS) in chloroplasts and oxidative stress^1^. ROS degrade natural metabolites through oxidative damage to fats, proteins, nucleic acids, enzymes and photosynthetic pigments^7^. Moghaddam et al. stated that irrigation treatment had remarkable effects on the plant biomass, number of capsules per plant, grain yield, grain oil content and harvest index of sesame^8^. In addition, irrigation regime could also affect seed oil composition^9–13^.

Arbuscular mycorrhizal fungi (AMF) have the most common and long-standing coexistence with almost 97% of plants and are essential factors in stable soil–plant systems^14^. The most important beneficial effect of AMF is the increase of host plant growth, usually by enhancing the uptake of immobile nutrients in the soil. AMF have been used in recent years to combat dehydration and drought stress in many plants^15^. Mycorrhizal coexistence also affects plant metabolism by increasing the concentration of metabolites in root and shoot^16^. One strategy to reduce the adverse effects of water stress on crop plants is the use of mycorrhizal fungi. Mycorrhizal inoculation can also increase oil percentage and oleic acid content in different plants^12^.

Phenols and flavonoids are the foremost compounds involved in the non-enzymatic antioxidant system and play important roles in controlling oxidative stress through scavenging reactive oxygen species^17, 18^. The effects of drought stress on phenolic compounds have been investigated in different crops^19^. For instance, drought condition enhanced the content of phenolic compounds in cumin (*Cuminum cyminum* L.) seed^9^. Under adverse environmental conditions, phenol production is activated due to the key roles of phenols in the plant defense system^20^. Besides, phenolic compounds have some anti-inflammatory and oxidative effects and are useful for the activation of the transcription factor Nrf2. Nrf2 plays an important role in cellular resistance to oxidative stress^21^. Sesame seeds contain a vast array of phenolic compounds including caffeic, ferulic, p-coumaric, and ellagic acids, having important biological and antioxidant properties^12, 18, 22^.

Despite extensive research on the coexistence of AMF with various crops, there is still limited information on the symbiosis relation of sesame plants with these fungi under drought stress. Unlike the previous studies that have focused more on the effects of one fungal species, the effects of different AMF species were investigated on the response of sesame cultivars to water stress in the present study. Yet, little is known about AMF potential effects on the quantity and the composition of sesame oil. The aim of our study was therefore to determine the effects of the inoculation by four AMF species on seed oil concentration and composition, of sesame plants grown under water stress condition.

## Materials and methods

### Experimental design and treatments

**Figure 1:**
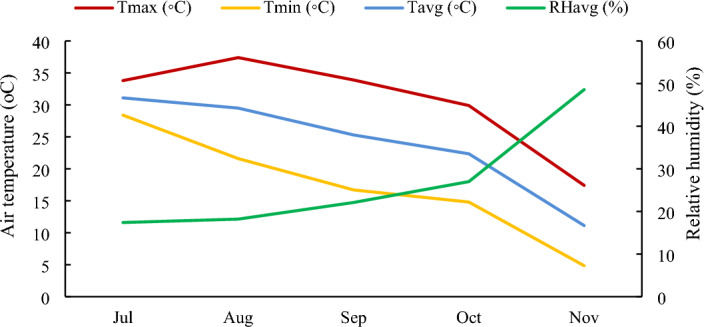
Minimum (Tmin), maximum (Tmax), and average (Tavg) air temperature, and average relative humidity (RHavg) during the growing season in 2019.

We collected a population of seeds from two cultivars grown at Lavark Reasrch Farm, Isfahan, Iran (32°32′N, 51°23′E, 1630 m a. s. l) with a mean annual precipitation of 140 mm. Minimum, maximum, and average air temperatures and average relative humidity are recorded in Fig. 1. In collection of seed material, we complied with our relevant institutional and national standards, and also with international guidelines and legislation complied with the IUCN Policy Statement on Research Involving Species at Risk of Extinction and the Convention on the Trade in Endangered Species of Wild Fauna and Flora. Some important soil physicochemical ‏characteristics are shown in Table 1. Treatments included inoculation by different mycorrhizal species (non-inoculated, *Funneliformis mosseae*, *Rhizophagus irregularis, Glomus fasciculatum,* and *Claroideoglomus claroideum*), two irrigation regimes (well-watered and water stress conditions) and two sesame cultivars (Yekta and Naz). This research was conducted as a factorial experiment in a randomized complete block design with three replications for each treatment. Each plot contained four rows, 2 m in length and 50 cm in width. Spacing between plants in the same row was 10 cm. All parameters were determined on the samples taken at full flowering stage.

**Table 1:** The characteristics of the original soil used in the experiment.

Parameter	Value
Textural class	Clay loam
Ec (dS m^−1^)	1.2
pH	7.3
Organic matter (%)	0.6
Total nitrogen (%)	0.08
Available P^a^ (mg kg^−1^)	21.6
Exchangeable K^b^ (mg kg^−1^)	380

^a^Olsen available.

^b^Available potassium (Kavail).

For mycorrhizal inoculation, 10 g (50–60 spores per g) of mycorrhizal inoculum (Touran Biotech Company, Shahrod, Iran) was added into the hole below each seed and the holes were concealed with the soil. For none-inoculated plants sesame seeds were sown with no inoculation.

### Application of irrigation treatments

Irrigation was performed based on 50% management allowed depletion (MAD) in full irrigation treatment. The maximum allowed soil moisture depletion (SMD) from the sesame root zone depth was calculated from the following equation:

1 $${\text{SMD }} = \, \left( {\uptheta_{{{\text{FC}}}} - \, \uptheta_{{{\text{PWP}}}} } \right) \, \times {\text{ MAD }} \times {\text{ Zr}}$$

where SMD is soil moisture depletion (mm), θ_FC_ is soil moisture content at field capacity (%), θ_PWP_ is soil moisture content at withering point (%), MAD is management allowed depletion for sesame is 0.5^23^, and Zr is root zone depth for sesame in this study equal 500 mm.

Calculated sesame evapotranspiration (ET_C_) was used to determine how much soil moisture was extracted from the root zone depth. The reference crop evapotranspiration (ET0) was calculated using FAO Penman–Monteith (FPM) method^23^ based on daily weather data from the Najafabad meteorological station (http://www.najafabadmet.ir/). Daily (ET_C_)i was calculated using ET0 and the site-specific sesame crop coefficient (K_C_) by below equation:

2 $$\left( {{\text{ETc}}} \right){\text{i }} = \, \left( {{\text{K}}_{{\text{C}}} *{\text{ ET}}0} \right){\text{i}}$$

where ET_C_ is daily sesame evapotranspiration (mm/day), ET0 is reference crop evapotranspiration (mm/day), and K_C_ is crop coefficient for each day during the growing stages, i is indicator for days. The sesame Kc for total growing season was extracted by using Kc.initail = 0.35, Kc.mid = 1.1, and Kc.lat = 0.25^23^.

The cumulative ETc values were calculated after each irrigation, and when ${\sum }_{i=1}^{n}\left(ETc\right)i\approx SMD$, the irrigation time was determined and the irrigation depth was equal to SMD, i is indicator for days.

To apply the stress treatment the irrigation interval was increased so that in the water stress treatment the irrigation interval was twice of the full irrigation treatment, but the applied irrigation depth for both full irrigation and the stress treatments was equal to SMD.

Irrigation was performed with a drip-tape irrigation system. Crop rows were irrigated with drip-tape (Tiran Fitting Polymer Co., Tiran, Isfahan, Iran) with drippers spaced 20 cm apart and 1.5 L h^−1^ flow rate for each dripper. For each irrigation treatment there was separated manifold pipe and valve to control irrigation amount and time. As the irrigation system was designed and implemented, the water pressure remained constant throughout the system. The irrigation was started on the first day after planting. All experimental treatments were irrigated simultaneously from sowing to before sesame flowering. Prior to flowering (about 40 days after planting) and until physiological maturity of the plant, irrigation levels were applied.

### Root mycorrhizal colonization

The percentage of root mycorrhizal colonization was determined using fresh roots. For this purpose, root tissue samples (with three technical replicates within each biological replicate for each treatment) were cleaned with KOH (10% w/v) at 95 °C for 1 h and the tissue was then acidified with HCl (1% w/v) for 1 h before staining in 0.05% lactic acid-glycerol-Trypan Blue^24^. Finally, the gridline intersection method of Giovannetti and Mosse was used to quantify the percentage of root colonization under light microscopy^25^.

### Oil concentration

For this purpose, the sesame seeds (three biological replicates for each treatment) were dried in the shade at room temperature for 3 weeks. The dried seeds powder (20 g) was added to 250 mL of petroleum ether to determine the oil concentration. Finally, oil concentration was extracted using a soxhlet extractor for five h according to the method reported by Jensen^26^.

### Determination of total phenolic and flavonoid concentrations

Total phenolic concentration (TPC) was assessed according to the Folin–Ciocalteu colorimetric method, as described by Ghaffari et al., with some modifications^27^. Briefly, 2 g of the sesame seed powder (three biological replicates for each treatment) was extracted in 10 mL of 80% methanol for 24 h at 25 °C. An aliquot of 500 µg of the methanolic extract solutions was mixed with 5 mL tenfold diluted Folin–Ciocalteu reagent (1:10 Folin–Ciocalteu, distilled water) and 4 mL of 7.5% sodium carbonate. The extract solutions were heated for 15 min at 45 °C and absorbance was read at 765 nm. Finally, the TPC was expressed as tannic acid equivalent per gram dry weight of the sample.

Total flavonoid concentration (TFC) in sesame seed samples were determined using UV spectrophotometry^28^. In summary, 125 µl of the aliquot of extract solution was added to 75 μL of a 5% NaNO_2_ solution. After 6 min, AlCl3 solution (10%, 150 μl) was added and allowed to stand for another 5 min before NaOH solution (1 M, 750 µl) was added. To this mixture distilled water was immediately added to bring the final volume to 2.5 mL. The obtained mixture was adequately mixed and allowed to stand for 15 min. Absorbance was determined at 510 nm using UV–visible spectrophotometry. The TFC in the extract was demonstrated as quercetin equivalent (mg QE/g dry mass).

### Total antioxidant activity

The stable purple-colored 2,2-diphenyl-1-picrylhydrazyl (DPPH) was used to determine the total antioxidant activity of sesame seeds according to Braca et al.^29^. Briefly, 50 µL of sesame seed extracts (three biological replicates for each treatment) were prepared in methanol (80%) before the DPPH solution (0.1 mM, 50 µL) was mixed with 100 μL of the diluted extracts. Ascorbic acid was employed as a positive control. The obtained mixture was vigorously mixed on a shaker and stored for 30 min at 37 °C in the dark. The absorbance of the resulting solutions was measured at 560 nm on a UV–visible spectrophotometer. Finally, total antioxidant activity was calculated by using the following equation:

3 $${\text{Scavenging effect }}\left( \% \right) \, = {1}\, - \,{\text{A sample}}\,{\text{A control}}\, \times {1}00$$

### Chromatographic separation of phenolic and flavonoid components using HPLC analysis

Phenolic and flavonoid components of sesame seeds were separated by an HPLC evaluation method described by Lin and Harnly with some modifications^30^. In brief, 2.5 g of sesame seeds (three biological replicates for each treatment) were powdered and extracted in 50 mL of methanol/water (80:20, v/v) for 18 h (110 rpm). Liquid chromatography was then performed on an Agilent Series 1090 HPLC system. Before injection into the analytical HPLC system, all the standards obtained from Sigma Co. were dissolved in HPLC-grade methanol (0.2–20 mg L^−1^). Finally, phenolic and flavonoid components of sesame seed extracts were expressed based on the peak areas and retention time of each one relative to those of the pure standards.

### GC analysis and identification of the fatty acid composition

To determine the fatty acid composition of sesame oil, the samples of sesame seed oils (three biological replicates for each treatment) were initially homogenized with vortex and 50 μl from each sample was taken. The oil was dissolved in 1 ml of n-hexane, and then 100 μl of methanolic sodium methoxide (0.5 N) was added. All the standards including palmitic acid, stearic acid, oleic acid, linoleic acid, and linolenic acid were dissolved in HPLC-grade methanol before injection into the analytical column for analysis. An SP-3420A, gas chromatography apparatus (SP-3420A, Beijing Beifen-Ruili Analytical Instrument, China) equipped with an FID detector was utilized for sample analysis. Separations were performed using an HP-88 capillary column, 100 m length, 250 μm i.d., and the thickness of the stationary phase was 0.2 μm. The oven temperature was programmed as follows: 1 min at 175 °C, subsequently 2.5 °C min—1 to 240 °C, held for 24 min at 240 °C. Nitrogen was used as the carrier gas with a flow rate of 1.1 mL min—Temperatures of the injection port and detector were set at 250 °C. The injector was set in a split mode (split ratio of 1:50) with an injection volume of 1 μl^31^.

### Statistical analysis

Data were assessed for normality and, when necessary, log-transformation was used before analysis, using the Shapiro–Wilk test. Data were subjected to analysis of variance (three-way ANOVA) and where treatment was statistically significant at P < 0.05, the least significant difference (LSD) test was used to detect specific differences. SAS statistical software version 9.1 (SAS Institute, Cary, NC, USA) was used for data analysis. The purpose of principal component analysis (PCA) was to reduce the number of measured variables on sesame and to measure multivariate co-linearity among the traits and items which was performed using StatGraphics (ver. 9.3).

## Results

### Root colonization

**Figure 2:**
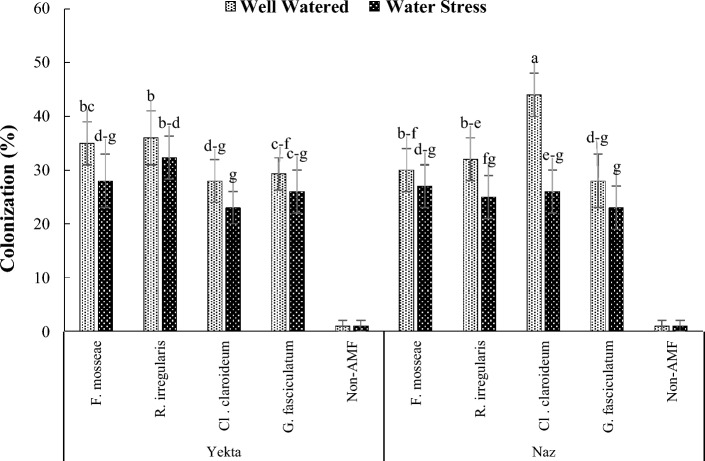
Effect of water regime and mycorrhizal fungi inoculation on mycorrhizal colonization of two sesame cultivars. Vertical bars (mean ± SD) not connected with the same letter represent significant difference between treatments according to LSD test (P < 0.05).

Root colonization was reduced by water stress in Yekta and Naz cultivars 14.8 and 24.6%, respectively (Fig. 2). The highest and the lowest decreases in Yekta (20 and 10.3%) were obtained in the plants inoculated by *F. mosseae* and *R. irregularis* mycorrhizal species and in Naz (41 and 10%) in the plants inoculated by *Cl. claroideum* and *F. mosseae*. The highest root colonization was observed in Naz plants inoculated by *Cl. claroideum* (44%) under well-watered condition but under water stress it was archived in Yekta plants inoculated by *R. irregularis* (32.3%). No AMF colonies were found on the none-inoculated plants roots (Fig. 2).

### Seed yield

Seed yield was significantly influenced by cultivar, irrigation regime, mycorrhiza and their interactions (Table 2). Seed yield of both tested cultivars was decreased by water stress under all inoculation treatments. The highest decrease (56.4%) was observed in Yekta inoculated by *R. irregularis* and the lowest one (24.4%) belonged to Naz inoculated by *Cl. claroideum* (Table 2). On the other hand, inoculation with mycorrhiza increased seed yield and the highest increase (59.8%) was observed in Naz inoculated by *Cl. claroideum* and the lowest one (3.77%) was obtained in Yekta inoculated by *Cl. claroideum* both grown under water stress. The maximum seed yield (3510 kg/ha) was achieved in Yekta plants inoculated by *R. irregularis* under normal irrigation and the lowest one (1240 kg) was obtained in none-inoculated plants of Naz under water stress condition.

**Table 2:** The effects of water regime and mycorrhizal fungi inoculation on seed yield, oil concentration, total flavonoid concentration (TFC), total phenolic concentration (TPC), and radical scavenging activity (RSA) of two sesame cultivars.

Cultivar	Fungi	Seed yield (kg ha^−1^)		Oil concentration (%)		TFC (mg g^−1^)		TPC (mg g^−1^)		RSA (mg g^−1^)	
		WW	WS	WW	WS	WW	WS	WW	WS	WW	WS
Yekta	*F. mosseae*	2786 ± 148^ed^	1616 ± 80^ij^	52 ± 2.57^a^	47.4 ± 2.50^be^	13.5 ± 1.10^i-k^	19.9 ± 1.58^d-f^	25.1 ± 1.88^g^	46.7 ± 2.35^b^	112 ± 8^ef^	126 ± 8^ef^
	*R. irregularis*	3510 ± 101^a^	1530 ± 110^j^	47.5 ± 2.3^ab^	45 ± 2.26^cd^	18.3 ± 1.67^e-g^	24 ± 2.18^b^	37.3 ± 2.40^de^	47.4 ± 1.68^ab^	136 ± 10^ef^	138 ± 7^ef^
	*Cl. claroideum*	2620 ± 105^c-e^	1293 ± 111^k^	47.9 ± 2.4^bc^	36.3 ± 1.45^e^	16.3 ± 1.44^gh^	29.5 ± 2.66^a^	30.2 ± 2.86^f^	50.2 ± 0.96^a^	110 ± 4^ef^	244 ± 6.02^c^
	*G. fasciculatum*	2590 ± 130^ef^	1750 ± 125^i^	48.4 ± 3.4^b^	34.4 ± 1.65^e-g^	11.5 ± 0.98^kl^	26.3 ± 1.89^b^	24.1 ± 1.45^gh^	49.2 ± 1.77^ab^	107 ± 12^ef^	142 ± 9^e^
	Non-AMF-inoculated	2426 ± 87^f^	1240 ± 58.6^k^	42.9 ± 2.85^d^	32.2 ± 1.60^f-h^	10.2 ± 0.9^l^	18.3 ± 1.25^e-g^	19.1 ± 0.85^i^	40.7 ± 1.98^c^	101 ± 12^f^	106 ± 18.7^ef^
Naz	*F. mosseae*	3220 ± 95^b^	1640 ± 121^ij^	50.2 ± 1.46^ab^	37.2 ± 1.40^e^	16.9 ± 2.15^gh^	20.5 ± 0.56^de^	36.2 ± 2.54^e^	49.3 ± 1.27^ab^	193 ± 8^d^	289 ± 9^ab^
	*R. irregularis*	2753 ± 105^c-e^	1700 ± 110^ij^	49.5 ± 2.3^ab^	31.2 ± 1.76^gh^	15.3 ± 1.59^hi^	21.2 ± 1.34^cd^	38.7 ± 2.42^ce^	47.8 ± 2.08^ab^	202 ± 9^d^	284 ± 10^ab^
	*Cl. claroideum*	2606 ± 120^d-f^	1970 ± 161^h^	43.5 ± 1.35^d^	35.1 ± 1.20^ef^	12.5 ± 0.96^j-i^	23.7 ± 2.22^bc^	29.1 ± 2.24^f^	49.4 ± 1.44^ab^	184 ± 7.50^d^	320 ± 10^a^
	*G. fasciculatum*	2800 ± 120^c^	1560 ± 120^j^	49.8 ± 2.1^ab^	34.1 ± 1.20^e-g^	15.1 ± 1.84^h-j^	18.9 ± 1.46^d-g^	28.6 ± 1.89^f^	47.1 ± 2.44^ab^	133 ± 6.45^cf^	277 ± 10.5^bc^
	Non-AMF-inoculated	2170 ± 100^g^	1233 ± 110^k^	34.7 ± 2.5^ef^	30.4 ± 1.60^h^	11.5 ± 0.87^kl^	17.4 ± 1.18^f-h^	21.2 ± 1.42^hi^	39.5 ± 1.60^cd^	128 ± 9.20^ef^	190 ± 5^d^

Source of variation	df	Seed yield, F.(P)	Oil concentration, F.(P)	TFC, F.(P)	TPC, F.(P)	RSA, F.(P)					
Cultivar (C)	1	10.26 (0.042)*	8.55 (0.039)*	12.9 (0.001)**	10.9 (0.002)**	20.9 (0.000)**					
Fungi (F)	4	14.4 (0.000)**	13.4 (0.000)**	8.63 (0.009)**	7.15 (0.009)**	15.4 (0.000)**					
Irrigation (I)	1	12.7 (0.004)**	5.21 (0.001)**	10.2 (0.000)**	12.7 (0.002)**	12.5 (0.049)*					
C × F	4	12.8 (0.001)**	7.50 (0.009)**	16.1 (0.003)**	12.4 (0.009)**	5.97 (0.003)**					
C × I	1	10.96 (0.034)*	9.81 (0.009)**	13.6 (0.001)**	17.1 (0.001)**	8.71 (0.005)**					
F × I	4	11.4 (0.009)**	17.2 (0.002)**	12.5 (0.000)**	18.4 (0.002)**	9.64 (0.001)**					
C × F × I	4	13.1 (0.001)**	13.5 (0.003)**	18.8 (0.004)**	7.22 (0.032)*	6.71 (0.009)**					
Error	36	–	–	–	–	–					

Means followed by similar letters within each column do not express significant differences (LSD test).

WW, well-watered; WS, water stress.

*Indicates significant at P < 0.05 and. **indicates significant at P < 0.01. df, degree of freedom. F and P are the values of Fisher table and probability level, respectively.

### Oil concentration

Seed oil concentration was significantly influenced by cultivar, irrigation regimes, mycorrhiza and their interactions (Table 2). Water stress decreased oil concentration and the maximum (36.9%) and the minimum (5.26%) decreases were belonged to Naz and Yekta plants inoculated by *R. irregularis* (Table 2). However, mycorrhizal inoculation increased oil concentration and the highest increase (47.2%) was achieved in Yekta plants inoculated by *F. mosseae* and the lowest one (2.63%) was observed in Naz plants inoculated by *R. irregularis*, both cases took place under water stress. Oil concentration was highest (52%) in Yekta inoculated by *F. mosseae* grown under well-watered condition and it was lowest (30.4%) in the seeds of Naz none-inoculated plants grown under water stress.

### Oil composition

The analysis of variance showed that cultivar, AMF, irrigation level, and their interactions significantly affected most of the oil composition components (Table 3). Water stress in most cases increased the contents of oleic, palmitic and stearic acids (Table 3). The highest increase in oleic acid in Yekta was achieved in the plants inoculated by *F. mosseae* (4.25%) and in none- inoculated plants of Naz (4.41%). The maximum increase in palmitic acid in Yekta was obtained in the plants inoculated by *Cl. claroideum* (12.4%) and in Naz in *G. faciculatum* inoculated (12.4%) and also in the none- inoculated plants (12.4%). The highest increase in stearic acid in Yekta (15.6%) and Naz (11.3%) belonged to none- inoculated plants. However, water stress decreased the contents of linoleic and linolenic acids under all inoculation treatments in both cultivars. The maximum decreases in linoleic (6.31%) and linolenic (60.3%) acids in Yekta were achieved in none- inoculated plants. The maximum decrease in linoleic acid (6.81%) in Naz was obtained in none-inoculated plants but the highest decrease in linolenic acid (53.8%) in Naz was obtained in the plants inoculated by *R. irregularis* (Table 3).

**Table 3:** The effects of water regime and mycorrhizal fungi inoculation on saturated and unsaturated fatty acids of two sesame cultivars.

Cultivar	Fungi species	Oleic acid (%)		Linoleic acid (%)		Linolenic acid (%)		Palmitic acid (%)		Stearic acid (%)	
		WW	WS	WW	WS	WW	WS	WW	WS	WW	WS
Yekta	*F. mosseae*	47 ± 2^g-i^	49 ± 1.2^c-e^	39.2 ± 1.2^a-c^	37.4 ± 0.4^ef^	0.498 ± 0.01^cd^	0.408 ± 0.008^ef^	7.26 ± 0.06^c-g^	7.22 ± 0.05^d-g^	6.03 ± 0.27^e-h^	5.72 ± 0.45^kl^
	*R. irregularis*	47.3 ± 0.8^f-i^	48.5 ± 1^d-g^	39.2 ± 0.4^a-c^	37.5 ± 0.9^ef^	0.590 ± 0.07^b^	0.368 ± 0.01^ fg^	7.11 ± 0.39^f-h^	7.43 ± 0.07^cd^	5.79 ± 0.1^i-k^	6.21 ± 0.05^c-e^
	*Cl. claroideum*	46.7 ± 1^i^	48.5 ± 1^d-g^	40 ± 0.9^ab^	37.8 ± 1^e^	0.497 ± 0.005^cd^	0.477 ± 0.007^cd^	7.07 ± 0.07^gh^	7.95 ± 0.1^ab^	5.74 ± 0.05^kl^	6.48 ± 0.08^b^
	*G. fasciculatum*	47.9 ± 1.2^e-i^	48.5 ± 0.85^d-g^	39 ± 0.4^b-d^	37.4 ± 0.5^ef^	0.568 ± 0.02^b^	0.492 ± 0.004^cd^	7.06 ± 0.05^gh^	7.33 ± 0.11^c-e^	5.54 ± 0.45^l^	6.21 ± 0.04^c-e^
	Non-AMF-inoculated	46.9 ± 1^g-i^	46.3 ± 0.3^i^	38 ± 1^de^	35.6 ± 1^hi^	1.15 ± 0.17^a^	0.456 ± 0.006^de^	7.33 ± 0.1^c-e^	7.88 ± 0.5^b^	6.48 ± 0.08^b^	7.49 ± 0.05^a^
Naz	*F. mosseae*	51.2 ± 1^ab^	50.4 ± 0.7^a-c^	36.1 ± 1^gh^	34.9 ± 2^ij^	0.399 ± 0.01^ef^	0.372 ± 0.004^ fg^	7 ± 0.5^h^	7.44 ± 0.06^c^	5.98 ± 0.05^f-i^	6.17 ± 0.1^d-f^
	*R. irregularis*	48.3 ± 0.85^d-h^	48.9 ± 0.9^c-f^	40.1 ± 0.5^a^	37.5 ± 1.5^ef^	0.475 ± 0.005^cd^	0.219 ± 0.005^h^	7.23 ± 0.11^d-g^	7.31 ± 0.04^c-f^	5.83 ± 0.04^h-k^	5.95 ± 0.03^g-j^
	*Cl. claroideum*	46.8 ± 1^hi^	48.4 ± 1^d-h^	39 ± 1.5^b-d^	38.2 ± 0.7^c-e^	0.568 ± 0.003^b^	0.530 ± 0.002^bc^	7.19 ± 0.04^e-h^	7.37 ± 0.11^c-e^	6.12 ± 0.03^d-g^	6.27 ± 0.05^cd^
	*G. fasciculatum*	49.9 ± 0.8^a-d^	51.4 ± 1^a^	38 ± 0.4^de^	36.1 ± 1^gh^	0.380 ± 0.04^f^	0.307 ± 0.003^g^	7.01 ± 0.39^h^	7.88 ± 0.12^b^	5.63 ± 0.1^kl^	6 ± 0.02^f-h^
	Non-AMF-inoculated	47.6 ± 2^e-i^	49.7 ± 1.1^b-d^	36.7 ± 1.2^ fg^	34.2 ± 0.8^j^	0.500 ± 0.01^cd^	0.499 ± 0.013^cd^	7.25 ± 0.06^c-g^	8.15 ± 0.1^a^	5.76 ± 0.27^jk^	6.41 ± 0.04^bc^

Source of variation	df	Oleic acid, F.(P)	Linoleic acid, F.(P)	Linolenic acid, F.(P)	Palmitic acid, F.(P)	Stearic acid, F.(P)					
Cultivar (C)	1	16.8 (0.004)**	9.41 (0.002)**	14.9 (0.001)**	0.33 (0.567)	8.30 (0.003)**					
Fungi (F)	4	10.0 (0.009)**	8.30 (0.000)**	7.71 (0.001)**	21.4 (0.002)**	9.62 (0.009)**					
Irrigation (I)	1	15.2 (0.001)**	13.8 (0.001)**	21.1 (0.009)**	18.9 (0.000)**	13.9 (0.001)**					
C × F	4	4.23 (0.000)**	11.5 (0.004)**	5.26 (0.047)*	5.87 (0.041)*	8.58 (0.000)**					
C × I	1	0.10 (0.979)	0.19 (0.666)	7.45 (0.044)*	2.26 (0.141)	19.1 (0.001)**					
F × I	4	0.58 (0.678)	1.36 (0.267)	6.08 (0.032)*	10.5 (0.001)**	20.6 (0.001)**					
C × F × I	4	3.08 (0.028)*	1.51 (0.218)	8.95 (0.027)*	14.4 (0.000)**	10.8 (0.000)**					
Error	36	–	–	–	–	–					

Means followed by similar letters within each column do not express significant differences (LSD test).

WW, well-watered; WS, water stress.

*Indicates significant at P < 0.05 and.**indicates significant at P < 0.01. df, degree of freedom. F and P are the values of Fisher table and probability level, respectively.

Mycorrhizal inoculation in most cases increased the contents of oleic and linoleic acids in both cultivars but for the case of oleic acid this was more evident in Yekta and in the case of linoleic acid it was more marked in Naz. The maximum increase in oleic acid was obtained by *F. mosseae* inoculation under water stress in Yekta (5.83%) but under well-watered irrigation regime it was achieved in Naz (7.56%). The highest increase in linoleic acid was observed in the plants inoculated by *Cl. claroideum* under water stress in both Yekta (6.17%) and Naz (11.7%). However, mycorrhizal inoculation in most cases decreased the contents of linolenic, palmitic and stearic acids. The maximum decreases in linolenic acids were achieved in Yekta inoculated by *F. mosseae* (56.7%) and *Cl. claroideum* (56.8%) under well-watered irrigation regime. The highest decrease in palmitic acids was observed in Naz inoculated by *R. irregularis*. (10.3%) and the highest decrease in stearic acids belonged to Yekta plants inoculated by *F. mosseae* (23.6%) both under water stress condition.

### Total phenolic and flavonoid concentrations

The concentrations of total phenolic and flavonoids were significantly influenced by irrigation regime, mycorrhiza and their interactions (Table 2). Water stress increased total concentrations of phenolic and flavonoids (Table 2). The maximum increases were obtained for phenolic concentration (113%) in the none-inoculated plants of Yekta and for flavonoids concentration (128%) in Yekta plants inoculated by *G. fasciculatum*. The minimum increases were attained for phenolic concentration (23.5%) in Naz plants inoculated by *R. irregularis* and for flavonoids concentration (21.3%) in Naz plants inoculated by *F. mosseae*.

Mycorrhizal inoculation increased the concentrations of total phenolic and flavonoids. The highest increases in phenolic (95.7%) and flavonoid (79.4%) concentrations were obtained in the plants of Yekta inoculated by *R. irregularis* under normal irrigation and the lowest increase in phenolic concentration (14.7%) was attained in Yekta plants inoculated by *F. mosseae* but in flavonoid concentration (8.62%) it was detected in Naz plants inoculated by *G. fasciculatum* both happened under water stress. The highest total concentrations of phenolic (50.2 mg g^−1^) and flavonoid (29.5 mg g^−1^) were observed in the plants inoculated with *Cl. claroideum* under water stress and the lowest concentrations (19.1 and 10.2 mg g^−1^, respectively) were observed in the none- inoculated plants under well-watered condition both were noticed in Yekta.

### High performance liquid chromatography (HPLC) of phenolic and flavonoid components

The analysis of variance showed that cultivar, AMF, irrigation level, and their interactions significantly affected most of the phenolic and flavonoid components (Table 4). Clearly, HPLC analysis detected six phenolic components including chlorogenic, elagic, gallic, caffeic, p-coumaric, and ferulic acids and also three flavonoid components including rutin, quercetin, and apigenin. The results showed that under water stress condition the contents of chlorogenic, and caffeic acids were increased in all inoculation treatments of Yekta and Naz cultivars (Table 4). The highest increase in chlorogenic acid (188%) was obtained in the non-inoculated plants of Yekta while the maximum increase in caffeic acid (11.9%) was observed in Naz plants inoculated by *G. fasciculatum*. However, for the other phenolic and flavonoids compounds no constant trend was noticed and the response to water stress was largely dependent on the interaction of cultivar and the inoculation treatment.

**Table 4:** The effects of water regime and mycorrhizal fungi inoculation on phenolic and flavonoid components of two sesame cultivars.

Cultivar	Fungi	Chlorogenic acid (µg g^−1^)		Ellagic acid (µg g^−1^)		Gallic acid (µg g^−1^)		Caffeic acid (µg g^−1^)		P-coumaric acid (µg g^−1^)	
		WW	WS	WW	WS	WW	WS	WW	WS	WW	WS
Yekta	*F. mosseae*	18.1 ± 0.5^jk^	37.7 ± 0.6^c^	0 ± 0^g^	0 ± 0^g^	9.59 ± 0.09^b^	6.24 ± 0.03^g^	0 ± 0^l^	64.8 ± 1^e-g^	14.5 ± 0.5^e-g^	14.8 ± 0.7^d-f^
	*R. irregularis*	15.8 ± 0.5^mn^	19.4 ± 0.7^i^	0 ± 0^g^	13.9 ± 0.9^d^	10.3 ± 0.3^a^	6.96 ± 0.06^d^	63.2 ± 1^ij^	71.9 ± 0.7^b^	15.2 ± 0.2^d^	13.9 ± 0.6^h^
	*Cl. claroideum*	15.2 ± 0.7^n^	37.2 ± 0.4^cd^	0 ± 0^g^	13.5 ± 0.4^d^	5.86 ± 0.06^h^	6.65 ± 0.05^f^	0 ± 0^l^	64.7 ± 0.8^e-h^	14.4 ± 0.4^f-h^	14.4 ± 0.3^f-h^
	*G. fasciculatum*	15.6 ± 0.6^mn^	29.7 ± 0.6^f^	0 ± 0^g^	17.1 ± 0.6^a^	5.85 ± 0.03^h^	8.58 ± 0.04^c^	64.2 ± 1^f-h^	65.2 ± 0.9^de^	17.9 ± 0.7^b^	17.9 ± 0.5^b^
	Non-AMF-inoculated	16.6 ± 0.4^l^	47.9 ± 0.5^a^	0 ± 0^g^	12.3 ± 0.2^e^	4.35 ± 0.05^k^	3.44 ± 0.06^m^	61.8 ± 0.8^k^	64.7 ± 1^e-h^	14.2 ± 0.2^gh^	14.5 ± 0.5^e-g^
Naz	*F. mosseae*	18.6 ± 0.5^j^	36.9 ± 0.5^d^	0 ± 0^g^	0 ± 0^g^	5.01 ± 0.06^j^	3.38 ± 0.05^mn^	65.9 ± 0.9^d^	67.5 ± 0.5^c^	15.2 ± 0.4^d^	15.0 ± 0.2^de^
	*R. irregularis*	15.8 ± 0.6^mn^	28.6 ± 0.2^g^	0 ± 0^g^	13.4 ± 0.6^d^	6.79 ± 0.04^e^	3.04 ± 0.15^o^	62.4 ± 1^jk^	65.0 ± 2^d-f^	18.1 ± 0.6^b^	16.7 ± 0.5^c^
	*Cl. claroideum*	16.2 ± 0.2^lm^	25.0 ± 0.5^h^	0 ± 0^g^	14.9 ± 0.7^c^	1.30 ± 0.15^q^	5.97 ± 0.04^h^	0 ± 0^l^	82.6 ± 0.6^a^	17.1 ± 0.25^c^	22.1 ± 0.6^a^
	*G. fasciculatum*	17.8 ± 0.5^k^	31.6 ± 0.4^e^	0 ± 0^g^	11.5 ± 0.5^f^	5.65 ± 0.3^i^	3.28 ± 0.03^n^	63.9 ± 1^g-i^	71.5 ± 1^b^	0 ± 0^i^	17.9 ± 0.5^b^
	Non-AMF-inoculated	16.1 ± 0.5^lm^	43.7 ± 0.3^b^	13.6 ± 0.4^d^	16.3 ± 0.7^b^	3.72 ± 0.09^l^	2.52 ± 0.24^p^	62.1 ± 0.4^k^	63.8 ± 0.8^hi^	14.4 ± 0.2^f-h^	15.1 ± 0.4^d^

Source of variation	df	Chlorogenic acid, F.(P)	Ellagic acid, F.(P)	Gallic acid, F.(P)	Caffeic acid, F.(P)	P-coumaric acid, F.(P)					
Cultivar (C)	1	7.26 (0.044)*	12.7 (0.000)**	15.5 (0.001)**	14.8 (0.004)**	0.01 (0.940)					
Fungi (F)	4	12.9 (0.009)**	14.0 (0.009)**	21.1 (0.004)**	14.9 (0.000)**	19.4 (0.001)**					
Irrigation (I)	1	18.7 (0.009)**	15.1 (0.000)**	14.6 (0.001)**	18.2 (0.003)**	7.95 (0.005)**					
C × F	4	20.6 (0.003)**	14.3 (0.001)**	6.82 (0.003)**	17.5 (0.009)**	8.67 (0.009)**					
C × I	1	7.46 (0.027)*	6.31 (0.002)**	0.61 (0.440)	10.4 (0.008)**	6.92 (0.001)**					
F × I	4	10.6 (0.004)**	18.6 (0.006)**	20.3 (0.000)**	9.25 (0.004)**	4.23 (0.002)**					
C × F × I	4	21.3 (0.000)**	12.1 (0.000)**	11.7 (0.007)**	14.4 (0.001)**	9.31 (0.000)**					
Error	36	–	–	–	–	–					

Cultivar	Fungi	Ferulic acid (µg g^−1^)		Rutin (µg g^−1^)		Quercetin (µg g^−1^)		Apigenin (µg g^−1^)			
		WW	WS	WW	WS	WW	WS	WW	WS		
Yekta	*F. mosseae*	47.1 ± 1^c^	45.2 ± 1.2^d^	62.5 ± 0.5^k^	70.6 ± 0.6^g^	0 ± 0^f^	30.7 ± 0.3^a^	0 ± 0^c^	42.3 ± 0.4^b^		
	*R. irregularis*	41.7 ± 0.5^hi^	41.6 ± 0.7^hi^	63.9 ± 0.7^j^	60.6 ± 0.3^m^	0 ± 0^f^	29.1 ± 0.6^c-e^	43.3 ± 0.6^a^	42.2 ± 0.4^b^		
	*Cl. claroideum*	41.7 ± 0.7^hi^	42.3 ± 1^g^	58.2 ± 0.4^p^	61.3 ± 0.7^l^	0 ± 0^f^	29.4 ± 0.4^bc^	0 ± 0^c^	42.1 ± 0.1^b^		
	*G. fasciculatum*	41.9 ± 0.9^gh^	51.8 ± 0.5^a^	57.9 ± 0.3^p^	61.5 ± 0.5^l^	0 ± 0^f^	28.8 ± 0.3^de^	42.3 ± 1^b^	41.9 ± 0.7^b^		
	Non-AMF-inoculated	0 ± 0^j^	41.4 ± 0.4^i^	59.3 ± 0.3^o^	59.3 ± 0.3^o^	0 ± 0^f^	0 ± 0^f^	0 ± 0^c^	0 ± 0^c^		
Naz	*F. mosseae*	44.6 ± 0.6^e^	42.0 ± 1^gh^	60.0 ± 1^n^	63.6 ± 0.4^j^	0 ± 0^f^	29 ± 1^c-e^	0 ± 0^c^	42.6 ± 1^ab^		
	*R. irregularis*	45.2 ± 0.2^d^	43.9 ± 0.6^f^	85.9 ± 0.3^c^	73.6 ± 0.5^f^	0 ± 0^f^	29.1 ± 0.4^c-e^	0 ± 0^c^	41.9 ± 0.6^b^		
	*Cl. claroideum*	45.4 ± 0.4^d^	45.0 ± 1^de^	104 ± 1^b^	74.8 ± 0.4^e^	28.7 ± 0.5^e^	29.3 ± 0.3^b-d^	0 ± 0^c^	42.4 ± 1^b^		
	*G. fasciculatum*	42.3 ± 0.5^g^	48.1 ± 0.6^b^	113 ± 0.8^a^	67.9 ± 0.9^h^	0 ± 0^f^	29.8 ± 0.6^b^	0 ± 0^c^	42.3 ± 0.8^b^		
	Non-AMF-inoculated	42.3 ± 1^g^	41.8 ± 0.8^hi^	66.3 ± 0.4^i^	77.3 ± 0.3^d^	0 ± 0^f^	0 ± 0^f^	0 ± 0^c^	0 ± 0^c^		

Source of variation	df	Ferulic acid, F.(P)	Rutin, F.(P)	Quercetin, F.(P)	Apigenin, F.(P)						
Cultivar (C)	1	9.83 (0.002)**	8.91 (0.004)**	10.5 (0.002)**	4.96 (0.000)**						
Fungi (F)	4	7.51 (0.000)**	7.55 (0.004)**	6.91 (0.005)**	9.25 (0.001)**						
Irrigation (I)	1	8.33 (0.001)**	9.73 (0.002)**	5.75 (0.002)**	4.37 (0.004)**						
C × F	4	6.64 (0.001)**	5.24 (0.001)**	11.2 (0.005)**	3.01 (0.002)**						
C × I	1	8.51 (0.005)**	9.62 (0.000)**	10.7 (0.001)**	5.12 (0.007)**						
F × I	4	9.14 (0.003)**	6.93 (0.001)**	6.54 (0.004)**	4.35 (0.001)**						
C × F × I	4	6.94 (0.000)**	7.09 (0.002)**	11.1 (0.000)**	3.79 (0.005)**						
Error	36	–	–	–	–						

Means followed by similar letters within each column do not express significant differences (LSD test).

WW, well-watered; WS, water stress.

*Indicates significant at P < 0.05 and. **indicates significant at P < 0.01. df, degree of freedom. F and P are the values of Fisher table and probability level, respectively.

Mycorrhizal inoculation in most cases decreased the content of chlorogenic acid and this was much more evident under water stress, so that the highest decrease in chlorogenic acid (59.5%) was attained under water stress condition in Yekta plants inoculated by *R. irregularis*. However, mycorrhizal inoculation in most cases increased the contents of gallic, caffeic, p-coumaric, and ferulic acids. There appeared no overall dominant trend in terms of rutin response to mycorrhizal inoculation. Yet, under water stress condition rutin content was increased in Yekta but it was decreased in Naz by all inoculation treatments.

The highest level of chlorogenic acid was achieved in the Yekta none-inoculated plants under water stress and that of gallic acid was observed in Yekta plants inoculated by *R. irregularis* under normal irrigation The maximum levels of caffeic and p-coumaric acids were observed in Naz plants inoculated with *Cl. claroideum* under water stress condition. The highest level of Ferulic acid belonged to Yekta plants under water stress and that of rutin compound belonged to Naz plants under normal irrigation, both groups were inoculated by *G. fasciculatum*.

### Total antioxidant activity

Total antioxidant activity was significantly influenced by cultivar, irrigation regime, mycorrhiza and their interactions (Table 2). Water stress increased total antioxidant activity and the highest increase (121%) belonged to the plants inoculated by *Cl. claroideum* and the lowest one (1.47%) was attained under *R. irregularis* inoculation both cases occurred in Yekta plants (Table 2). Mycorrhizal inoculation also increased total antioxidant activity (Table 2). The maximum increase (130%) was achieved in the plants of Yekta inoculated by *Cl. claroideum* under water stress and the lowest one (3.91%) was observed in the plants inoculated by *G. fasciculatum,* under normal irrigation both belonged to Naz plants. The highest total antioxidant activity (320 mg g^−1^) belonged to Naz plants inoculated by *Cl. claroideum* under water stress condition and the lowest activity (101 mg g ^−1^) was observed in Yekta none- inoculated plants under normal irrigation.

### Principle component analysis (PCA)

**Figure 3:**
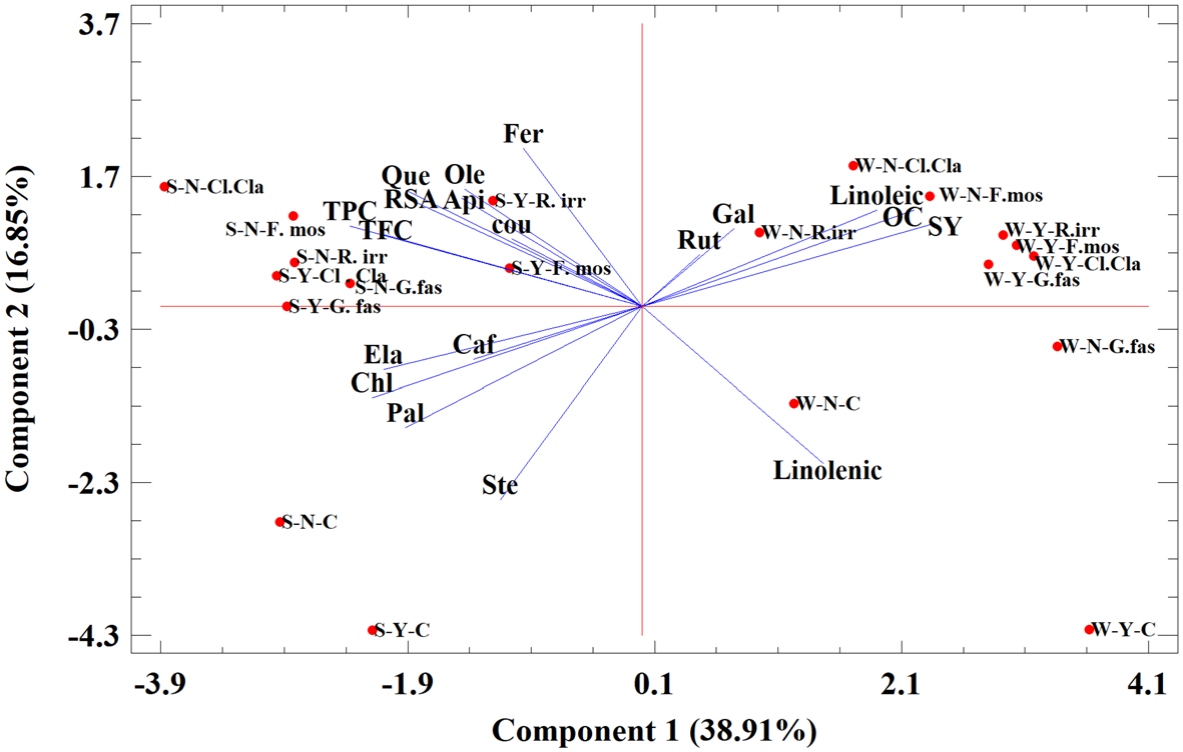
Principal Component Analysis (PCA) performed on characteristics of two sesame cultivars inoculated with mycorrhizal fungi. N (Naz); Y (Yekta); W: well-watered; S: water stress; *Rhizophagus irregularis* (R.irr); *Funneliformis mosseae* (F.moss); *Claroideoglomus claroideum* (Cl.cla); *Glomus fasciculatum* (G.fas); C: Non-inoculated plants; Fer: ferulic acid; Chl: chlorogenic acid; Caf: caffeic acid; Ela: elagic acid; Gal: gallic acid; p-coumaric, Rut: rutin; Que: quercetin; Api: apigenin; Ole: oleic acid; Pal: palmitic acid; Ste: stearic acid; SY: Seed yield; OC: oil content; TPC: Total phenolic concentration; TFC: total flavonoid concentration; RSA: radical scavenging activity.

The results of biplot analysis showed that the first two principal components together explained for more than 50% (51.8%) of the variability among the traits (Fig. 3). The treatments defined in the experiment were scattered in all four quarters of the biplot; however, the largest number of treatments were placed in the B and D biplot quarters so that all the non-stress and control treatments were on the right side of the biplot and all stress treatments were located on the left side of the biplot; therefore, PCA was able to completely separate the two groups of stress and non-stress treatments, while the two cultivars were not separated from each other by the principal components method. The non-AMF conditions versus the use of different types of AMF were somewhat differentiated, a way that the non-AMF treatments were placed in the lower part of the biplot (A and C) with negative PC2 values.

The relationship between traits based on the vector angels in biplot showed that grain yield in both cultivars had its highest value in control conditions and had a high and positive correlation with oil concentration and grain linoleic acid content, while other grain properties such as antioxidant activity and flavonoid concentration were not correlated with grain yield. About phenolic compounds, the increase of compounds is mainly observed under stress condition, especially for chlorogenic acid, ellagic acid and caffeic acid which were increased under stress condition in both cultivars.

## Discussion

In this study, water stress decreased mycorrhizal root colonization but the reduction was smaller in Yekta cultivar and also in the plants inoculated by *G. fasciculatum*. The significant interaction of irrigation level and mycorrhizal species on the mycorrhizal colonization was also reported by Aalipour et al.^14, 32^. They reported that the highest and the lowest root colonization respectively belonged to the *F. mosseae* and *R. irregularis* species under water stress condition^14^.

Water deficit also reduced sesame seed yield and oil concentration and the minimum decreases in seed yield were obtained in the plants inoculated by *G. fasciculatum* (24%) and *Cl. claroideum* (32%) but for oil concentration the lowest decreases were observed in the plants inoculated by *R. irregularis* (5%) and in the non-inoculated plants (12%) of Naz and Yekta cultivars, respectively. It is known that mycorrhizal species vary in terms of their effects on seed yield and oil concentration^34^. For example, Hamedani et al. reported that among different mycorrhizal species used for sesame plants, *F. mosseae* had the highest effects on the increasing of seed yield and oil content^33^. Also, Gholinezhad et al. stated the superiority of *F. mosseae* over *R. intraradices* in terms of positive effects on sesame seed yield^34^.

Mycorrhizal inoculation improves the nutritional, biochemical, physiological and morphological conditions of the host plant, thus it increases plant resistance to biotic and abiotic stresses, including water stress^32, 35^. The positive effects of mycorrhizal coexistence on improving water relations and host plant resistance to drought stress have been proven and several mechanisms have been proposed to justify this situation. The most important of these mechanisms is the direct absorption of water and its transmission through the fungal hyphae to the host plants^11^. Numerous studies have reported increased water use efficiency of the mycorrhizal inoculated plants under water stress^14, 36^.

Significant variation was observed among mycorrhizal species in terms of motivation of grain yield and alleviation of water deficit effects, as the highest increase in seed yield (60%) due to inoculation was observed in Naz plants inoculated by *Cl. Claroideum* under water stress condition. In the study of He et al. inoculation of mung bean plants with *G. mosseae* and *G. caledonium* under drought stress increased plant growth by 1.99 and 1.80 times, respectively^37^. Gholinezhad and Darvishzadeh^38^, reported that sesame grain yield was increased by 25 and 33% due to *G. intraradices* and *G. mosseae* inoculation, respectively. This was related to the positive effects of mycorrhizal inoculation on phosphorus uptake, longer leaf shelf life, maintaining and increasing leaf size and also improving photosynthesis by retaining higher chlorophyll content^39^. In the study of Koocheki et al., inoculation with *G. mosseae* and *G. intraradices* improved growth, water use efficiency and physiological characteristics of sesame but to a different degree^10^. Kyriazopoulos et al. stated that inoculation with *R. intradadices* increased biomass production in *Dactylis glomerata* more than that in *F. mosseae*^40^. Hamedani et al. reported that inoculation by *F. mosseae* as compared to *R. intradadices* had greater effects on grain yield and seed oil content of sesame under drought stress^33^.

The positive interaction of mycorrhiza with oil plants has been reported as an important factor helping plants to protect them from various stresses^41^. Several researchers have investigated the potential of AMF to promote the accumulation of bioactive compounds in oil plants^11–13^. Gholinezhad and Darvishzadeh investigating the effects of two AMF species on grain yield and oil content of 8 local sesame cultivars and reported that AMF inoculation, especially with *G. mosseae*, improved grain yield and quality traits of sesame seeds under drought stress and well-watered conditions^38^. Igiehon et al. found that the content of fatty acids in seeds of soybean plants were increased by mycorrhizal inoculaion under drought stress^13^. Rahimzadeh and Pirzad also reported that higher oil and mucilage percentage of the flaxseed (*Linum usitatissimum* L.) plants were obtained from AMF inoculated plants^11^. They also reported that plants inoculated with AMF promoted benefits on the α-linolenic, linoleic and oleic acid synthesis. AMF inoculation could also be considered as a sustainable strategy based on natural resources in order to positively influence the oil content of flaxseed^11^.

Differential response of fatty acid components to water stress was observed in the current study, as water stress in most cases increased the contents of oleic (unsaturated), palmitic (saturated) and stearic acids (saturated) but decreased linoleic (unsaturated) and linolenic (unsaturated) acids. These results, therefore, show that, except oleic acid, water stress largely tends to reduce unsaturated but to increase saturated fatty acids contents. Accordingly, in the study of Tohidi Moghadam et al. water stress decreased the growth period and sink capacity and thus limited the time to produce unsaturated fatty acids in canola grains^42^. However, in contrast, Ali et al. reported that water stress had adverse effects on saturated fatty acids of *Celosia argenteaan* but it presented positive effects on monounsaturated and polyunsaturated fatty acids^43^.

These results confirm the findings of previous research that reported increased oleic, palmitic and stearic acids but decreased linoleic and linolenic acids under water stress conditions^44, 45^.

However, in contrast with the response to water stress, except for linolenic, mycorrhizal inoculation in most cases increased the content of unsaturated (oleic and linoleic) but decreased the content of saturated (palmitic and stearic) fatty acids. This trend is consistent with the findings of Gholinezhad and Darvishzadeh, who showed that AMF inoculation increased the levels of oleic and linoleic acids and reduced erucic acid concentration in sesame grains. Consequently, inoculation of plants by mycorrhiza is expected to increase grain oil quality^12^. It is known that D-12 desaturase and D-9 desaturase are the key enzymes involved in oleic acid synthesis^46^. Therefore, the hypothesis of the present study is that the positive effect of AMF inoculation on unsaturated fatty acids especially oleic acid might be due to the activation of these two enzymes. Nevertheless, the physiological and biochemical bases for the role of AMF on fatty acids composition needs to be better clarified.

Coincidence increases were evident in TPC and TFC concentrations and total antioxidant activity by water stress. TPC and TFC are important for acclimation and adaptive responses of plants to environmental stresses‏^47^. In fact, the activation of phenylalanine ammonia-lyase (PAL), cinnamate 4-hydroxylase (C4H), chalcone synthase (CHS), and 4-coumarate CoA ligase (4CL), is associated with the accumulation of phenolic and flavonoid compounds^48, 49^.

Antioxidant activities were also increased in the mycorrhizal inoculated plants. As inoculated plants produced higher TPC and TFC under both well-watered and drought conditions, the higher antioxidant activities in the inoculated plants would be explained by the presence of these compounds which have strong antioxidant activity^50^. Previous studies have also shown increases in TPC and TFC in sesame plants by different mycorrhizal species^12, 22^. Gholinezhad and Darvishzadeh^12^, Kermani et al.^22^, and Taghizadeh et al.^51^, reported an elevation in total phenol content when sesame plants were inoculated with mycorrhiza. In fact, mycorrhiza motivates the accumulation of phenylalanine ammonia-lyase which is the key enzyme for the biosynthesis of phenolic compounds^52^. In the present investigation, higher TPC and TFC in mycorrhizal inoculated plants might, therefore, be a key factor for the improvement of sesame growth under drought condition.

In this study six phenolic (chlorogenic, elagic, gallic, caffeic, p-coumaric, and ferulic acids) and three flavonoid (rutin, quercetin, and apigenin) components were detected by HPLC analysis. The highest contents of caffeic, p-coumaric and rutin were observed in Naz but the highest contents of chlorogenic, gallic and ferulic acids were attained in Yekta, indicating genotype dependency of the accumulation of these compounds in sesame plants.

Significant variation existed among TPC and TFC components in response to water stress. While the contents of chlorogenic, and caffeic acids in all inoculation treatments and cultivars were increased by water stress, no general pattern over treatments and cultivars was observed regarding the response of the other compounds to water stress. Also, similar to our findings Nouraei et al. showed that chlorogenic and caffeic acid contents were increased during water shortage^53^.

Mycorrhizal inoculation in most cases decreased chlorogenic acid but increased gallic, caffeic, p-coumaric, and ferulic acids while changes were dependent on mycorrhizal species and irrigation regime. No foremost trend was seen for rutin in this regard. Accordingly, in the study of Alizadeh et al. inoculation with two mycorrhizal species (*F. mosseae* and *C. etunicatum*) influenced the amount of phenolic compounds in *Dracocephalum moldavica* L^54^. They reported that, the concentrations of p-coumaric, chlorogenic, and caffeic acids were increased under AMF inoculations. They also found that the amounts of gallic acid and rutin were decreased by *F. mosseae* strain but they were increased by *C. etunicatum* strain^54^. In our study also the influence of AMF inoculation on TPC and TFC components was dependent on the used mycorrhizal species, as the highest amounts of caffeic and P-coumaric acids in Naz cultivar belonged to the plants inoculated with *Cl. claroideum* strain, but in Yekta the highest amounts of these compounds were observed in the plants inoculated with *R. irregularis* and *G. fasciculatum* respectively, both cases occurred under water stress condition (Table 4).

This study showed that water stress as compared to mycorrhizal inoculation was a more prominent discriminator of treatments. In fact, cultivars inoculated with different mycorrhiza species under water stress were grouped together characterized by higher concentrations of phenolic and flavonoid concentrations while the same treatment under non-stress condition featured higher seed yield and oil concentration. Therefore, although inoculation with all forms of mycorrhiza may improve seed and oil yields of sesame, a lower stress condition for maximizing these traits in the two cultivars with seemingly low tolerance to water deficit is recommended.

## Conclusions

In summary, significant interactions existed in terms of the effects of water stress and mycorrhizal inoculation on seed yield and oil concentration and composition of two sesame cultivars Naz and Yekta. Water stress largely tended to reduce unsaturated but increased saturated fatty acids content. In contrast, mycorrhizal inoculation in most cases increased unsaturated while decreased saturated fatty acids. The simultaneous increases in total phenolic, flavonoid and antioxidant activity by water stress and inoculation showed the importance of these compounds in adaptation to biological and environmental factors. PCA analysis revealed that seed yield in both cultivars had its highest value in control condition and had a high and positive correlation with oil concentration. Also, among the phenolic compounds, the only compound in which the amount was increased in Naz cultivar under control condition was caffeic acid. While in the other phenolic compounds, the increase was mainly observed under water stress condition in both cultivars. In conclusion, it was observed that the two cultivars showed similar physiological and biochemical response to AMF inoculation under water stress condition, although the response was somewhat stronger in Naz as compared to Yekta cultivar.

## Data Availability

All data generated and analyzed during this study are included in this paper.
